# Impact of a voluntary industry code for advertising food to children and young people: an analysis of New Zealand television data

**DOI:** 10.1017/S1368980021004705

**Published:** 2022-05

**Authors:** Stephanie Shen, Sally Mackay, Arier Lee, Cliona Ni Mhurchu, Ahmed Sherif, Helen Eyles

**Affiliations:** 1 Department of Epidemiology and Biostatistics, School of Population Health, The University of Auckland, Auckland, New Zealand; 2 Department of Pacific Health, School of Population Health, The University of Auckland, Auckland, New Zealand

**Keywords:** Food, Policy, Children, Young people, Television, Advertising

## Abstract

**Objective:**

To evaluate the impact of the 2017 update to the voluntary Advertising Standards Authority (ASA) code for advertising food on children and young people’s exposure to unhealthy food advertisements on New Zealand television.

**Design:**

Audience ratings data were analysed for New Zealand children and young people’s television viewing for eight random days prior to (June to August 2015) and following (October to December 2018) the code update, from 06.00 to midnight (864 h). Food advertisements were coded using three nutrient profiling models. The number of children and young people watching television each year was compared.

**Setting:**

Three free-to-air New Zealand television channels.

**Participants:**

New Zealand children aged 5–18 years.

**Results:**

Television viewer numbers decreased over the 3 years (*P* < 0·0001). The mean rate of unhealthy food advertising on weekdays was 10·4 advertisements/h (2015) and 9·5 advertisements/h (2018). Corresponding rates for weekend days were 8·1 and 7·3 advertisements/h, respectively. The percentage of food advertisements which were for unhealthy foods remained high (63·7 % on weekdays and 65·9 % on weekends) in 2018. The ASA definition of children’s ‘peak viewing time’ (when 25 % of the audience are children) did not correspond to any broadcast times across weekdays and weekend days.

**Conclusions:**

Between 2015 and 2018, children and young people’s television exposure to unhealthy food advertising decreased. However, almost two-thirds of all food advertisements were still unhealthy, and the updated ASA code excluded the times when the greatest number of children was watching television. Consequently, government regulation and regular monitoring should reflect the evolving food marketing environment.

New Zealand has the second highest child obesity prevalence of all forty-one countries in the Organisation for Economic Co-operation and Development and European Union (behind the USA)^(1)^. The most recent New Zealand Health Survey in 2019/2020 revealed the obesity prevalence for children aged 2–14 years was 9·4 % (79 000 children)^(2)^. The same survey reported a greater prevalence of childhood obesity for Pacific (29·1 %) and Māori (indigenous population; 13·2 %) children, compared with 7·2 % of European/Other children^(2)^. Obesity is a form of malnutrition and the consequences of childhood obesity can extend into adulthood, with the development of life-limiting co-morbidities such as diabetes, osteoarthritis, CVD and cancers^(3,4)^.

Several systematic reviews have affirmed unhealthy food advertising as a risk factor for childhood obesity, observed through children’s food intake and increased positive attitudes towards advertised foods^(5–7)^, and these early unhealthy eating patterns are likely to continue into adulthood^(8)^. In 2010 at the 63rd World Health Assembly, the WHO introduced a Set of Recommendations for the Marketing of Foods and Non-alcoholic Beverages to Children^(9)^. The twelve recommendations relate to forming policies to restrict marketing of unhealthy foods to children, and their implementation was highlighted in the 2016 WHO Ending Childhood Obesity report^(4)^. In 2018, the WHO policy recommendations were also recognised in a document developed by the United Nations Convention on the Rights of the Child to promote child rights-based approaches to protect children from harmful content, including unhealthy food marketing^(10)^.

Governments and food and marketing organisations from several countries have responded with policies to reduce and regulate children’s exposure to unhealthy food marketing^(11–13)^, usually taking one of three forms: mandatory (the policy is passed by the government (legislation) and must be followed by all food companies), self-regulatory (the policy is managed by the food and marketing industry and company participation is voluntary)^(14)^ and co-regulatory (the policy is a combination of government and self-regulatory initiatives). Previous systematic reviews^(15–17)^ suggest mandatory policies are marginally more successful than self-regulatory policies, and countries such as Chile where mandatory controls are in place have seen a reduction in children’s exposure to unhealthy food advertising^(18,19)^.

In New Zealand, the industry-led Advertising Standards Authority (ASA) oversees a self-regulatory code to protect children from unhealthy food advertising in all virtual (e.g. television, websites, apps) and physical settings that target children or young people (except product packaging, and within news and broadcast programmes). In October 2017, a revised Children and Young People’s Advertising Code came into effect after recommendations from an independent review, replacing two previous codes related to food advertising to children^(20,21)^. The revision was informed by consultation with the public and public health stakeholders to reflect the goal of adequately protecting children (under 14 years) and young people (14–18 years) from unhealthy food advertisements. Changes to the code are outlined in Table 1. In summary, a definition was added for ‘targeting children’ (advertisements intended for, or appealing to children)^(20)^, the Ministry of Health Food and Beverage Classification System from 2013 was added to define an occasional (unhealthy) food or beverage^(22)^, and ‘children as a significant proportion of the audience (25 % of the total audience), and ‘special duty of care’ (to ensure advertising is not likely to result in physical, mental or moral harm) were defined^(20,21)^.

**Table 1:** Summary of changes to the ASA children and young people’s advertising code

Definition	Before ASA code update^(21)^	After ASA code update in 2017
Advertisements ‘targeting’ children	No definition	Three criteria:^(20)^ ‘1. Nature and intended purpose of the product or service being promoted is principally or generally appealing to children or young people. 2. Presentation of the advertisement content (e.g. theme, images, colours, wording, music and language used) is appealing to children or young people. 3. Expected average audience at the time or place the advertisement appears includes a significant proportion of children or young people.’
Occasional food and beverage products	No classification system to define foods intended for occasional consumption	Defined by the 2013 Ministry of Health Food and Beverage Classification System^(22)^
‘Significant proportion’ of the average audience	No definition	Defined as 25 % of the total audience^(21)^. Occasional food and beverage advertisements are inappropriate for advertising to children when children are expected to be a significant proportion of the average audience
Special duty of care	No definition	Referenced in relation to unhealthy food advertising to young people, defined as a ‘responsibility to ensure advertising is not likely to result in physical, mental or moral harm^(20)^’. However, there are no examples to explain this definition

After the announcement of the code changes in 2017, over seventy New Zealand public health professionals published a letter stating that the proposed changes were unlikely to make a significant impact on reducing children’s exposure to unhealthy food marketing^(23)^.

A study assessed unhealthy food advertising on New Zealand television before the code update in 2017. The study found that the previous self-regulatory code failed to protect children from unhealthy food advertising as most unhealthy food advertisements were shown when the greatest number of children was watching television^(16,24)^. Two different nutrient profiling models (WHO-Europe nutrient profiling model (WHO-EU) and the Ministry of Health Food and Beverage Classification System from 2007 (MoH 2007)) were used to categorise unhealthy food advertisements on New Zealand television in 2015^(25)^, finding children were exposed on average to 9·1 unhealthy food advertisements per hour (under WHO-EU). WHO-EU was the strictest nutrient profiling model and classified more food advertisements overall as unhealthy compared with MoH 2007 (68·5 % *v*. 50·5 %, respectively)^(24)^.

However, there have been no studies to determine the effectiveness of the 2017 revised ASA code. The aim of this study was to evaluate the impact of the 2017 ASA code update on the number of unhealthy food advertisements on New Zealand television and children’s exposure to such advertisements using television and child audience data before (2015) and after (2018) the code update.

## Methods

Methods were informed by the 2015 evaluation of the New Zealand ASA code by Vandevijvere *et al*.^(24)^ and to align with the protocol for such analyses published by the International Network for Food and Obesity/non-communicable diseases Research, Monitoring and Action Support (INFORMAS) group^(26)^. The primary outcome was to compare the number of children exposed to unhealthy food advertisements on television before and after the New Zealand ASA code update. Other outcomes were the mean rate and percentage of food advertisements that were unhealthy, and children as a proportion of total audience watching television in 2018. We also assessed whether the definition of children’s peak viewing times in the revised code (when 25 % of the audience are children) corresponded to when most children were watching television.

### Television channels, years, hours and days

The three most popular free-to-air channels in New Zealand were the same in 2015 and 2018; these were ONE, TV2 and TV3, which were rebranded in 2018 to TVNZ1, TVNZ2 and THREE, respectively.

In both years, television recordings were collected for eight random days (four weekdays and four weekend days) using the University of Auckland’s satellite recording service, UniSat. School holiday periods and public holidays were excluded. The hours of recording were from 06.00 to midnight, totalling 864 h across both years. The months recorded each year (July to August in 2015 and October to December in 2018) were different because there were no recordings available from July to August in 2018 due to the limited storage time of the UniSat recordings.

### Number of children watching television

Data on audience ratings (number of New Zealand children watching a certain television channel per half-hour of the day) for the three television channels were purchased from Nielsen^(27)^. Audience rating data were reported daily from 06.00 to midnight. Two Excel spreadsheets were obtained for the years 2015 and 2018 detailing audience ratings for weekdays, and weekends, over eight sampled days (four weekdays and four weekend days). Information on television watching by key ethnic groups in New Zealand, that is, Māori and Pacific, was not sufficient to assess (too few panellists), and thus these data were not able to be included. School holiday periods and public holidays were excluded. Mean audience ratings from the three channels were reported separately, and as combined totals across all channels (including channels not used for monitoring) for the age groups 5–13, 14–18 and 19+ years (the latter age group was included in 2018 only).

### Coding food advertisements

Television recordings from each year were viewed and coded by two different coders in 2015 and 2018. Data were collected for each advertisement and collated in a Microsoft Excel spreadsheet. A ‘food advertisement’ was defined as any advertisement containing food and/or drinks, as well as advertisements for food retailers such as supermarkets and food outlets (even if no specific food or beverages were shown). Advertisements containing food products or food companies/brands (e.g. supermarkets) were further coded where possible to comply with the INFORMAS protocol for television food advertising monitoring, including the placement (country, day, date, channel, time of broadcast), descriptive information about the food or beverage company/brand and types of promotional or premium strategies used. The coding from each year was cross-checked by each coder and reviewed again before data analysis to ensure the coding was consistent across years.

### Classification of food advertisements (healthy/unhealthy)

For 2015 data, advertisements which contained food products or food companies/brands were classified as healthy or unhealthy using two nutrient profiling systems: the WHO-Europe nutrient profiling model (WHO-EU; permitted/not permitted for marketing to children)^(28)^ and the New Zealand Ministry of Health Food and Beverage Classification System (MoH 2007; everyday, sometimes or occasional foods)^(25)^. The WHO-EU was the main classification system used to report unhealthy food advertisements in the study because it was specifically designed to restrict unhealthy food advertising to children and is recommended as the most appropriate and sensitive classification system to identify unhealthy foods. This nutrient model categorises foods based on threshold values for certain nutrients (energy, sugar, salt and/or fat)^(29)^. The MoH 2007 nutrient model was designed for guiding schools to sell healthier foods and drinks to children and categorises foods based on specific food examples and recommended serving sizes^(28)^. For 2018 data, food advertisements were classified using the two aforementioned models, as well as a third: the New Zealand Food and Beverage Classification System that was developed in 2013 and then updated in 2016 (MoH 2013)^(22)^. This third model, like WHO-EU, categorises foods based on certain nutrients and was important to include because the ASA currently classifies foods using these criteria^(20)^.

Food composition data for packaged foods were sourced from the New Zealand Nutritrack database, an annually updated brand-specific database of packaged foods sold at New Zealand supermarkets^(30)^. Food composition data for fast foods and restaurant foods were sourced directly from the company websites or the New Zealand Food Composition Database^(31)^.

### Data preparation and statistical analysis

The 2015 and 2018 coded datasets were analysed separately in Microsoft Excel 2013. Data from weekdays and weekends were also analysed separately. For each weekday and weekend, the total number of unhealthy food advertisements per half-hour was calculated using each nutrient profiling model (Table 2) and converted to an hourly rate. The number of unhealthy food advertisements was also calculated as a percentage of total food advertisements.

**Table 2:** Total and percentage of food advertisements and unhealthy food advertisements, and mean unhealthy food advertisements per hour (standard deviation, sd) on television in New Zealand, June–August 2015 and October–December 2018

	Weekdays								Weekends							
	2015				2018				2015				2018			
	Number	%	Mean	sd	Number	%	Mean	sd	Number	%	Mean	sd	Number	%	Mean	sd
Total advertisements	5738	100			8375	100			4733	100			7050	100		
Non-food advertisements*	4748	82·7			7305	87·2			3973	83·9			6250	88·7		
Food advertisements†	990	17·3			1070	12·8			760	16·1			800	11·3		
Unhealthy food ads (WHO-EU)	751	75·9	10·4	6·2	682	63·7	9·5	4·8	580	76·3	8·1	5·8	527	65·9	7·3	5·6
Unhealthy food ads (MoH 2007)	659	66·6	9·2	5·6	479	44·8	8·3	4·6	597	78·6	6·7	5·0	481	60·1	6·7	5·1
Unhealthy food ads (MoH 2013)	N/A‡	N/A‡	N/A‡	N/A‡	619	57·9	8·6	4·7	N/A‡	N/A‡	N/A‡	N/A‡	501	62·6	7·0	5·3

WHO-EU, WHO-Europe nutrient profiling model; MoH 2007, New Zealand Ministry of Health Food and Beverage Classification System 2007; MoH 2013, New Zealand Ministry of Health Food and Beverage Classification System 2013

* Includes alcohol and baby formula.

† Excludes alcohol and baby formula.

‡ The MoH 2013 nutrient profiling model was not applied to the 2015 data as it was not used in the study by Vandevijvere *et al*.^(24)^.

The total number of unhealthy food advertisements per half-hour in each weekday and weekend was graphed against time of the day (using a line plot) to illustrate the change in unhealthy food advertising throughout the day. On the same graph, the total audience ratings, to illustrate total exposure for 5–13 and 14–18 years, were plotted (as bars). Next, the percentage of 5–13 years watching television out of the total audience for each half-hour slot in 2018 (on weekdays and weekends separately) was calculated and graphed using a separate line plot. This was done to compare the percentage of viewers that were children at each time slot to compare with the 25 % significant viewing threshold indicated in the ASA code^(20)^.

Multiple linear regression was conducted to assess the association between exposure to unhealthy food advertising (by analysing the numbers of children and young people watching television) and year. Log transformation was applied to the number of children and young people watching television as viewing numbers were highly skewed. Multiple linear regression was conducted using SAS SAS/STAT software, Version 9.4 of the SAS System for Windows (SAS Institute Inc.).

## Results

### Number of children exposed to unhealthy food advertisements on television during weekdays

Overall, there were less children watching television in 2018 compared with in 2015 (*P* < 0·0001).

**Fig. 1:**
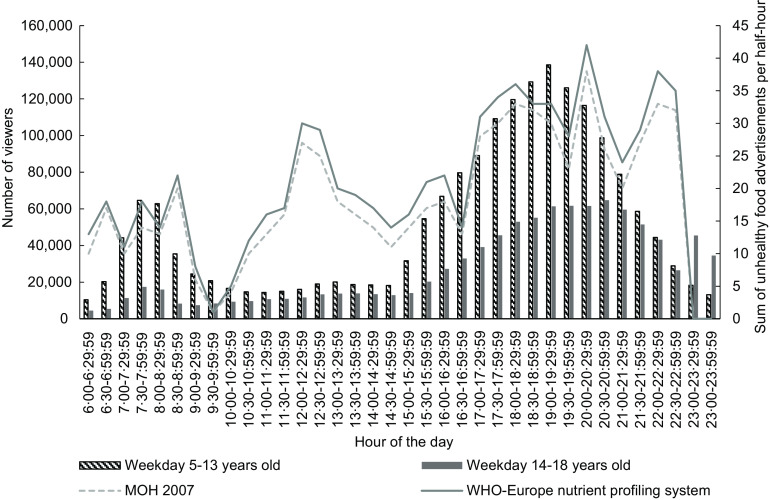
Weekday number of children (5–13 years) and young people (14–18 years) viewing television (bars) and total unhealthy food advertisements (lines) by half-hour time slot, New Zealand, June–August 2015, all television channels combined

**Fig. 2:**
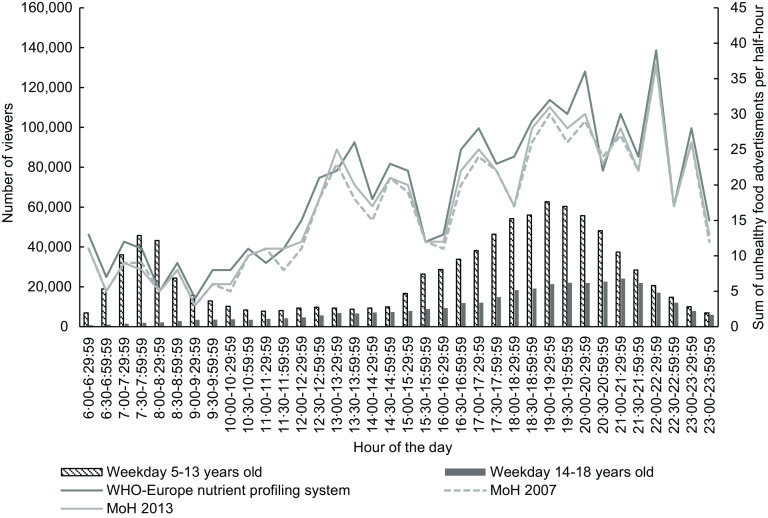
Weekday number of children (5–13 years) and young people (14–18 years) viewing television (bars) and total unhealthy food advertisements (lines) by half-hour time slot, New Zealand, October–December 2018, all television channels combined

Figures 1 and 2 illustrate the number of children aged 5–13 years and young people 14–18 years viewing television every half-hour (see bars) and trends in unhealthy food advertising (see line plots) during weekdays in 2015 and 2018, respectively. The patterns of children and young people’s television viewing and unhealthy food advertising across the day were similar for both years.

There was a peak number of children watching television in the morning (at 07.30–08.00 in 2015 and 2018) that then decreased during the day, before increasing to a second peak in the evening (19.00–19.30 in 2015 and 2018). The peak number of children watching television was lower in 2018 (*n* 45 700 in the morning and *n* 62 700 in the evening) than in 2015 (*n* 64 600 in the morning and *n* 138 600 in the evening). There were fewer young people watching television than children in both years. Young people’s viewing peaked in the evening at 21.00–21.30 in 2018 (*n* 24 200), but the total number was less than young people viewing at the peak time of 20.30–21.00 in 2015 (*n* 64 700).

The number of unhealthy food advertisements was low in the morning and increased during the day. The peak number of unhealthy food advertisements in the early afternoon (*n* 26 at 13.30–14.00) and evening (*n* 39 at 22.00–22.30) during 2018 was slightly lower than the peak numbers in 2015 (*n* 30 at 12.00–12.30 and *n* 42 at 20.00–20.30).

### Number of children exposed to unhealthy food advertisements on television during weekends

**Fig. 3:**
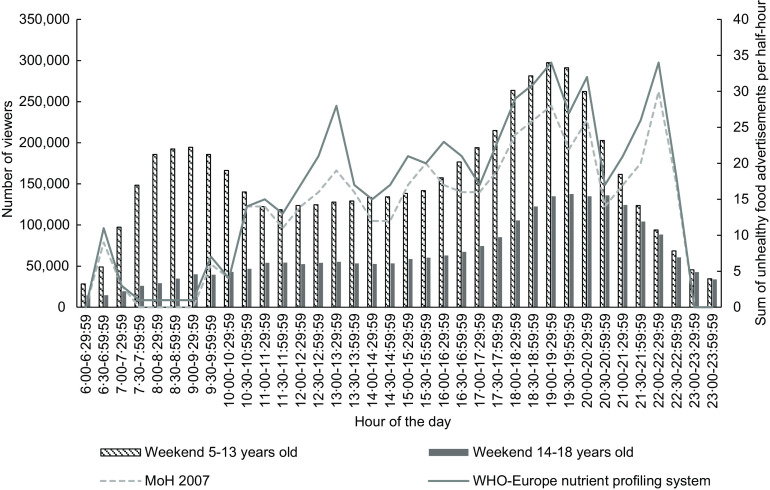
Weekend number of children (5–13 years) and young people (14–18 years) viewing television (bars) and total unhealthy food advertisements (lines) by half-hour time slot, New Zealand, June–August 2015, all television channels combined

**Fig. 4:**
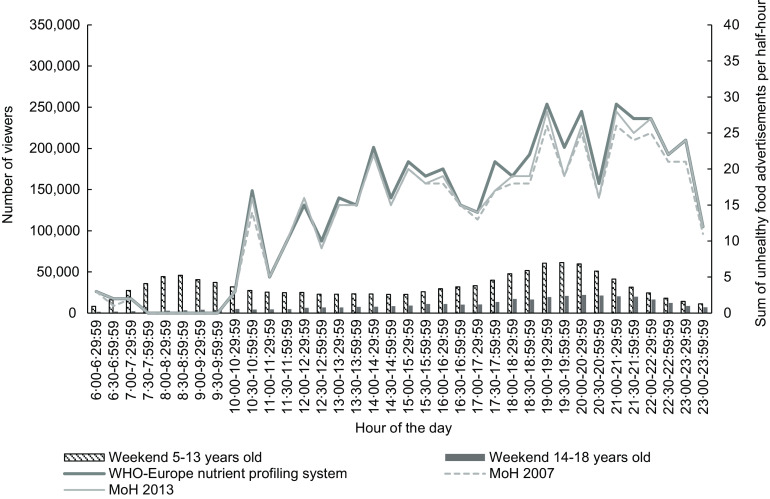
Weekend number of children (5–13 years) and young people (14–18 years) viewing television (bars) and total unhealthy food advertisements (lines) by half-hour, New Zealand, October–December 2018, all television channels combined

Figures 3 and 4 show the number of children aged 5–13 years and young people 14–18 years viewing television every half-hour (see bars) and trends in unhealthy food advertising (see line plots) during weekends in 2015 and 2018, respectively. The patterns of children and young people’s television viewing were similar in the weekends as weekdays.

There was a small peak of number of children watching television in the morning at 07.30–08.30 that dropped during the middle of the day, before increasing to a higher peak in the evening (19.00–19.30 in 2015 and 19.30–20.00 in 2018). There were low numbers of young people watching television throughout the day, which rose and peaked in the evening at around 19.30–20.00 in 2015 and 2018.

The trend for unhealthy food advertisements across the day appeared similar for both years, with the lowest number of unhealthy food advertisements seen in the morning which gradually increased throughout the day. There was a small peak in the early afternoon at 13.00–13.30 in 2015 and 14.00–14.30 in 2018. A drop in the evening took place in both years at the 20.30–21.00 slot and increased in the next half-hour time slot. In 2018, there were two peak time slots in the evening, where there were twenty-nine unhealthy food advertisements during 19.00–19.30 and 21.00–21.30. This was lower than the peak of thirty-four unhealthy food advertisements in 2015 during 22.00–22.30.

### Children as a proportion of total audience watching television in 2018

**Fig. 5:**
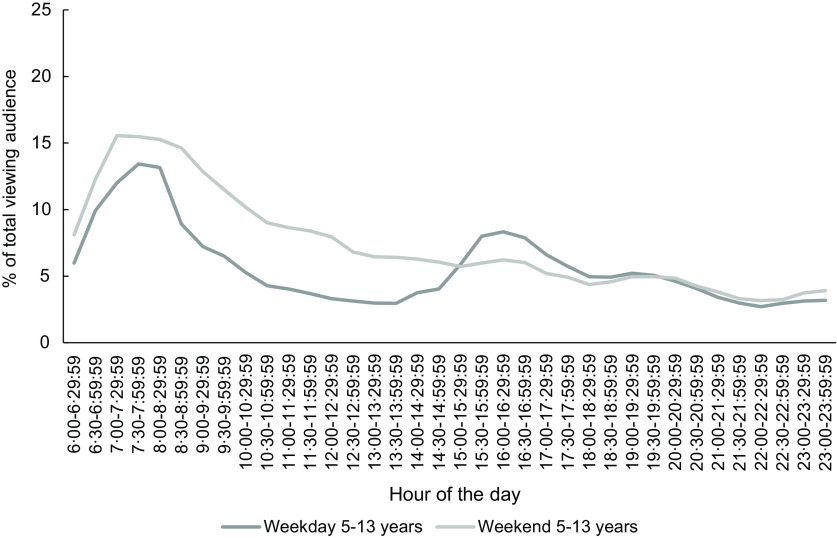
Percentage of 5–13 years watching television out of total viewing audience per half-hour during weekdays and weekends, October-December 2018, all television channels combined

Figure 5 shows children (5–13 years) as a proportion of all television viewers by half-hour time slots in 2018 during weekdays and weekends. Across all time slots, the percentage of the total audience who were children was lower than the ASA code’s peak viewing time definition of at least 25 % of the total viewing audience being children (less than 14 years). The percentage of the total audience who were children peaked early morning (6.30–8.00 at 13·4 %) and then dropped slowly over the day on both weekdays and weekends, with an exception of a second peak viewing time on weekdays only around 15.00–16.00.

### Mean rate and percentage of unhealthy food advertisements

Table 2 summarises the number of all advertisements, food advertisements and unhealthy food advertisements, as well as the mean hourly rate of unhealthy food advertisements by weekday and weekend day for each year.

The mean hourly rate of unhealthy food advertisements was lower in 2018 than in 2015 on weekdays (9·5/h (sd = 4·8/h) in 2018 *v*. 10·4/h (sd = 6·2/h) in 2015) and weekends (7·3/h (sd = 5·6/h) in 2018 *v*. 8·1/h (sd = 5·8/h) in 2015).

The percentage of unhealthy food advertisements relative to all food advertisements (as classified by the WHO-EU nutrient profiling model) decreased from 75·9 % on weekdays and 76·3 % on weekends in 2015 to 63·7 % on weekdays and 65·9 % on weekends in 2018 (Table 2).

## Discussion

This study was the first to assess the impact of the revised ASA industry code, a self-regulatory code to reduce unhealthy food advertising to New Zealand children. Between 2015 and 2018, the total number of unhealthy food advertisements overall decreased. This may have been due to a shift of unhealthy food advertisements to online marketing such as Facebook, where 98 % of food advertisements exposed to young people in New Zealand were unhealthy^(32)^. However, the proportion of all food advertisements on television that were unhealthy remained high. This suggests that the impact of the revised code is minimal as unhealthy foods continue to make up most food advertising on New Zealand television.

The results also highlight that television viewing decreased for children and young people between 2015 and 2018. Despite free-to-air television viewership decreasing, New Zealand’s overall screen time appears to be increasing due to the shift to other forms of media. This has been confirmed by other reports on audience behaviour which showed a decrease of television audiences from 83 to 66 % and increase of video streaming (from 6 to 37 %) and online video such as YouTube and Facebook (30–52 %) between 2014 and 2018^(33)^.

The current ASA code definition of children’s peak viewing time (25 % of the total viewing audience consisting of children up to 14 years) is an arbitrary threshold and in 2018 the percentage of child television viewers never came close to reaching the ASA definition. The proportions of children watching in New Zealand were similar to those in a South Australian report that found no more than 17 % of 0–14 years viewed television on weekdays, while a peak percentage of 0–14-year-old viewers (over 25 %) lasted for 3·5 h on weekend mornings^(34)^.

The evening times when children aged 5–13 years were the lowest percentage of the audience were also when the greatest absolute number of children and young people was watching television and the greatest number of unhealthy food advertisements was shown. Thus, the current ASA definition of peak viewing time does not reflect when the greatest number of children is watching, and exposed to, unhealthy food advertising on television.

The UK government recently responded with a ban on high fat, sugar and salt foods shown on live television and on-demand programmes (free-to-air and paid) before 21.00 and for paid-for advertising online from 2022^(13)^. Results from a modelling study estimated that the impact of banning high fat, sugar and salt foods from 05.30 to 21.00 on UK television would result in children viewing 1·5 fewer high fat, sugar and salt advertisements per day and reduce overweight and obesity by 3·6 %^(35)^. According to our New Zealand television analysis, a watershed period would be relevant until 22.00, when there are still at least 50 000 children and young people watching television.

Public health professionals have advocated for the use of the WHO-EU nutrient profiling model in New Zealand as it was specifically designed to restrict marketing of unhealthy food to children and was shown to be more sensitive than the Ministry of Health Food and Beverage Classification systems currently used by the ASA code. The latter was developed for classifying the healthiness of foods sold in schools^(23)^.

In 2020, the World Cancer Research Fund International released a report on the effectiveness of international policies to restrict inappropriate food advertising to children^(36)^. According to thirty-eight studies from eleven countries, which all used television data to assess impact, improvements to reduce unhealthy food advertising to children were seen in less than one quarter of the studies (8/38 studies). From this, 6/12 evaluations looked at mandatory policies (in Canada (Quebec), Chile, South Korea and the UK), which was more promising than the 2/26 evaluations of self-regulatory policies (in Australia and the USA). Similarly, three global systematic reviews also looked at both mandatory and self-regulatory policies and showed greater reductions in unhealthy food advertising and marketing from mandatory policies compared with self-regulatory policies^(15,17,37)^.

While mandatory policies are recommended by international public health agencies, most countries appear to be adopting self-regulatory policies due to food industry pressure and opposition^(38)^. But when assessed against a public health law framework^(39)^, the ASA code did not meet the benchmark for best regulatory practice and was deemed an ineffective policy to support a healthier food marketing environment^(40)^. Public health stakeholders are calling for movement towards mandatory government regulation to prevent further deviation from a children’s rights-based approach^(10,41,42)^. Other recommendations include involving multiple stakeholders during the policy development, separation of implementation and evaluation stages, and involving industry, government, civil society groups, public health officials and academics^(14)^. Stronger and more precise definitions are emphasised, for instance, to apply the definition of ‘children’ in the code to include up to 18 year olds in accordance with the United Nations Convention on the Rights of the Child^(42,43)^.

Currently, very few countries have regular practices to monitor and evaluate the effectiveness of policies to restrict unhealthy food and drink advertisements on television or other media over time, despite a recommendation from the WHO^(11,44,45)^. It is worthwhile to conduct research independently of the food and advertising industry to provide a universal benchmark for research when globally assessing policies over time^(16,26)^. The results from this study and subsequent monitoring of the impact from the 2017 ASA code update will further inform the changes needed to be made under the next review of the ASA code in 2022 to increase its impact^(46)^.

A strength of this study was that it was the first to use objective television programming and viewing data to assess the impact of changes to the New Zealand ASA code in 2017. It also compares data from before (2015) and after (2018) the code, which provides an indication of changes over time. Data from the two years were collected and analysed using the same protocol guided by INFORMAS, to ensure a consistent and validated methodology^(26)^. A further strength is that the data on the number of children and young people watching television from both 2015 and 2018 were purchased from Nielsen. These were comprehensive datasets collected from representative samples of New Zealanders^(27)^.

A limitation of this study was that it only looked at television data from a small number of free-to-air channels and did not include incidental television exposure, pay-to-view platforms, internet or social media, which are growing rapidly in popularity in New Zealand and globally^(47)^. The most recent Children’s Media Use Survey commissioned by NZ On Air and the Broadcasting Standards Authority in 2020 revealed that 35 % of children watched free-to-air television, compared with 73 % of children who watched video using overseas sites such as YouTube^(47)^. A further limitation was that, due to lack of sufficient audience viewing data, the study did not analyse Māori and Pacific audience data separately, which is important due to the disproportionately higher prevalence of childhood obesity in these groups^(2)^. As a result, the study could not examine whether viewing numbers and thus the impact of the code would be equal for different population groups.

The study also compared the different time periods in 2015 (July–August) and 2018 (October–December) which could have potentially different seasonal patterns. However, when comparing the audience rating data, daily trends did not seem to significantly differ, with peaks still taking place during the same times of the day.

As the ASA code applies to all advertising settings, future research should continue to monitor the New Zealand food advertising environment on all platforms to ensure the ASA code is a credible tool. The greatest challenge is creating a reliable methodology to measure the nature and extent of advertising exposure to children on web-based and social media platforms. Unlike television, internet and social media content is always accessible which inherently makes all children vulnerable to advertising at any time of the day. Barriers to monitoring include the ethical challenges of collecting personal data from social media users, and personalised tailoring of advertisements depending on individual use of media^(44,45,48)^. Options could be to use a browser extension to monitor advertisements seen online^(49)^ or to utilise a 2019 comprehensive monitoring framework developed by WHO^(45)^.

A final limitation was that there was no television audience data for children 0–5 years, an age group that is increasingly exposed to screens, as reported in the Growing Up in New Zealand study^(50)^. The data from this study indicated that at 2 years old, New Zealand children spend approximately 1·5 h each day on screens (including television and electronic media such as computer, laptop, tablets, smart phones and electronic gaming devices). This increases to 2 h per day at 3·75 years old^(50)^.

## Conclusion

We explored change over time in the number of unhealthy food advertisements New Zealand children were exposed to on television following the ASA code update in 2017. Since adoption of the revised ASA code, there has been a small reduction in unhealthy food advertisements on free-to-air television channels and fewer children and young people watching free-to-air television. However, two-thirds of food advertisements are still classified as unhealthy and children never make up 25 % of the total viewing audience, making the ASA definition of peak viewing time ineffectual. Our findings indicate the need for stronger regulation, increasing the code’s coverage to include children and young people up to 18 years, redefining ‘peak viewing time’ using an absolute number of children and young people rather than the arbitrary percentage of 25 % and the need to adopt a more stringent nutrient profiling model to define foods that are appropriate/not appropriate for marketing to children (WHO-Europe nutrient profiling model). Ultimately, government regulation and rigorous, independent monitoring of compliance across all media (including web-based and social media platforms) should be implemented to ensure protection of New Zealand children from unhealthy food marketing.

## References

[1] United Nations Children’s Fund (UNICEF) (2019) The State of the World’s Children 2019. Children, Food and Nutrition: Growing Well in a Changing World. UNICEF. https://www.unicef.org/media/60806/file/SOWC-2019.pdf (accessed December 2019).

[2] Ministry of Health (2020) Key Indicators. https://minhealthnz.shinyapps.io/nz-health-survey-2019-20-annual-data-explorer/_w_c4d95025/#!/key-indicators (accessed January 2021).

[3] Ng M , Fleming T , Robinson M et al. (2014) Global, regional, and national prevalence of overweight and obesity in children and adults during 1980–2013: a systematic analysis for the global burden of disease study 2013. Lancet 384, 766–781. 24880830 10.1016/S0140-6736(14)60460-8 PMC4624264

[4] World Health Organization (2016) Report of the Commission on Ending Childhood Obesity. Geneva: WHO.

[5] Boyland EJ , Nolan S , Kelly B et al. (2016) Advertising as a cue to consume: a systematic review and meta-analysis of the effects of acute exposure to unhealthy food and nonalcoholic beverage advertising on intake in children and adults. Am J Clin Nutr 103, 519–533. 26791177 10.3945/ajcn.115.120022

[6] Russell SJ , Croker H & Viner RM (2019) The effect of screen advertising on children’s dietary intake: a systematic review and meta-analysis. Obes Rev 20, 554–568. 30576057 10.1111/obr.12812 PMC6446725

[7] Qutteina Y , De Backer C & Smits T (2019) Media food marketing and eating outcomes among pre-adolescents and adolescents: a systematic review and meta-analysis. Obes Rev 20, 1708–1719. 31468652 10.1111/obr.12929

[8] Lipsky LM , Haynie DL , Liu D et al. (2015) Trajectories of eating behaviors in a nationally representative cohort of U.S. adolescents during the transition to young adulthood. Int J Behav Nutr Phys Act 12, 138. 26537771 10.1186/s12966-015-0298-x PMC4632654

[9] World Health Organization (2010) Marketing of Food and Non-Alcoholic Beverages to Children. Sixty-Third World Health Assembly. http://apps.who.int/gb/ebwha/pdf_files/WHA63/A63_R14-en.pdf (accessed July 2019).

[10] Garde A , Byrne S , Gokani N et al. (2018) A Child Rights-Based Approach to Food Marketing: a Guide for Policy Makers. Geneva. https://www.unicef.org/csr/files/A_Child_Rights-Based_Approach_to_Food_Marketing_Report.pdf (accessed December 2019).

[11] World Health Organization (2018) Evaluating Implementation of the WHO Set of Recommendations on the Marketing of Foods and Non-Alcoholic Beverages to Children: Progress, Challenges and Guidance for Next Steps in the WHO European Region. Copenhagen: WHO.

[12] World Health Organization (2019) Member States Consultation on the Draft Regional Action Framework on Protecting Children from the Harmful Impact of Food Marketing 2020–2030. Manila: WHO Regional Office for the Western Pacific.

[13] Powell T , Gheera M , Foster D et al. (2021) The Health and Care Bill (Bill 140 of 2021–22). House Commons Library. 61–69. https://researchbriefings.files.parliament.uk/documents/CBP-9232/CBP-9232.pdf (accessed July 2021).

[14] World Cancer Research Fund International (2020) Building Momentum: Lessons on Implementing Robust Restrictions of Food and Non-Alcoholic Beverage Marketing to Children. https://www.wcrf.org/sites/default/files/PPA-Building-Momentum-3-WEB-3.pdf (accessed February 2020).

[15] Galbraith-Emami S & Lobstein T (2013) The impact of initiatives to limit the advertising of food and beverage products to children: a systematic review. Obes Rev 14, 960–974. 23845093 10.1111/obr.12060

[16] Kelly B , Vandevijvere S , Ng SH et al. (2019) Global benchmarking of children’s exposure to television advertising of unhealthy foods and beverages across 22 countries. Obes Rev 20, 116–128. 30977265 10.1111/obr.12840 PMC6988129

[17] Chambers SA , Freeman R , Anderson AS et al. (2015) Reducing the volume, exposure and negative impacts of advertising for foods high in fat, sugar and salt to children: a systematic review of the evidence from statutory and self-regulatory actions and educational measures. Prev Med 75, 32–43. 25735606 10.1016/j.ypmed.2015.02.011

[18] Dillman Carpentier FR , Correa T , Reyes M et al. (2019) Evaluating the impact of Chile’s marketing regulation of unhealthy foods and beverages: preschool and adolescent children’s changes in exposure to food advertising on television. Public Health Nutr 23, 747–755. 31822317 10.1017/S1368980019003355 PMC7060093

[19] Jensen ML , Carpentier FD , Adair L et al. (2021) Examining Chile’s unique food marketing policy: TV advertising and dietary intake in preschool children, a pre- and post- study of Chile’s Food Marketing Policy. Int J Behav Nutr Phys Act 18, 1–11. 33947436 10.1186/s12966-021-01126-7 PMC8097821

[20] Advertising Standards Authority (2017) Children and Young People’s Advertising Code and Guidance Notes. http://www.asa.co.nz/wp-content/uploads/2017/02/ASA-Media-Release-Children-and-Young-Peoples-Advertising-Code-1-March-Website.pdf (accessed April 2019).

[21] Roy H (2017) Media Release: ASA Releases New Comprehensive Children and Young People’s Advertising Code. https://www.asa.co.nz/2017/03/01/new-comprehensive-children-young-peoples-advertising-code-released-today/ (accessed April 2019).

[22] Ministry of Health (2016) *Food and Beverage Classification System Nutrient Framework for Schools. Ministry of Health*. Wellington: Ministry of Health. https://www.asa.co.nz/wp-content/uploads/2017/02/FBCS-Nutrient-Criteria-March-2016.pdf (accessed December 2019).

[23] Swinburn B , Vandevijvere S , Woodward A et al. (2017) Proposed new industry code on unhealthy food marketing to children and young people: will it make a difference? N Z Med J 130, 94–101. 28207729

[24] Vandevijvere S , Soupen A & Swinburn B (2017) Unhealthy food advertising directed to children on New Zealand television: extent, nature, impact and policy implications. Public Health Nutr 20, 3029–3040. 28545596 10.1017/S1368980017000775 PMC10261618

[25] Ministry of Health (2007) *Food and Beverage Classification System for Years 1–13: User Guide. Ministry of Health*. Wellington: Ministry of Health. https://weightmanagement.hiirc.org.nz/assets/legacy/files/FBClassification/heha-user-guide-years1-13.pdf (accessed December 2019).

[26] Kelly B (2017) INFORMAS Protocol: Food Promotion Module: Food Marketing – Television Protocol. University of Auckland. https://figshare.com/articles/INFORMAS_Protocol_Food_Promotion_Module_Food_Marketing_-_Television_Protocol/5664706 (accessed August 2018).

[27] The Nielsen Company (2019) Television Audience Measurement. https://www.nielsen.com/nz/en/solutions/measurement/television/methodology/ (accessed August 2019).

[28] World Health Organization (2015) WHO Regional Office for Europe Nutrient Profile Model. http://www.euro.who.int/__data/assets/pdf_file/0005/270716/Nutrient-children_web-new.pdf (accessed January 2019).

[29] Ni Mhurchu C , Mackenzie T & Vandevijvere S (2016) Protecting New Zealand children from exposure to the marketing of unhealthy foods and drinks: a comparison of three nutrient profiling systems to classify foods. N Z Med J 129, 41–53. 27607084

[30] The National Institute for Health Innovation (2017) The Nutritrack Database. 1–2. https://diet.auckland.ac.nz/sites/default/files/2019-08/The_Nutritrack_Database.pdf (accessed January 2019).

[31] Ministry of Health & Plant & Food Research (2019) About. New Zealand Food Composition Data. https://www.foodcomposition.co.nz/about/ (accessed January 2019).

[32] Kidd B , Mackay S , Swinburn B et al. (2020) AdHealth: a feasibility study to measure digital food marketing to adolescents through Facebook. Public Health Nutr 24, 215–222. 32878674 10.1017/S1368980020001561 PMC10195416

[33] Glasshouse Consulting (2018) Where are the Audiences? New Zealand Air. https://d3r9t6niqlb7tz.cloudfront.net/media/documents/NZ_On_Air_May_2018_WHERE_ARE_THE_AUDIENCES_-_FINAL_for_print_and_web.pdf (accessed July 2019).

[34] Brindal E , Corsini N & Hendrie G (2011) Television Food Advertising to Children in South Australia. CSIRO. https://www.aph.gov.au/DocumentStore.ashx?id=b5f11e56-aa61-4ae1-bb2d-2d52710b3565 (accessed January 2020).

[35] Mytton OT , Boyland E , Adams J et al. (2020) The potential health impact of restricting less-healthy food and beverage advertising on UK television between 05.30 and 21.00 hours: a modelling study. PLOS MED 17, 1–22. 10.1371/journal.pmed.1003212 PMC7553286 33048922

[36] World Cancer Research Fund International (2020) Building Momentum Evidence Table: Effects of Implemented Restrictions on Food Advertising and other Forms of Commercial Promotion. https://www.wcrf.org/sites/default/files/BM3-evidence-table.pdf (accessed February 2020).

[37] Ronit K & Jensen JD (2014) Obesity and industry self-regulation of food and beverage marketing: a literature review. Eur J Clin Nutr 68, 753–759. 24713622 10.1038/ejcn.2014.60

[38] Swinburn B & Wood A (2013) Progress on obesity prevention over 20 years in Australia and New Zealand. Obes Rev 14, 60–68. 24102746 10.1111/obr.12103

[39] Reeve B & Magnusson R (2018) Regulation of food advertising to children in six jurisdictions: a framework for analyzing and improving the performance of regulatory instruments. Arizona J Int Comp Law 35, 71.

[40] Sing F , Mackay S , Culpin A et al. (2020) Food advertising to children in New Zealand: a critical review of the performance of a self-regulatory complaints system using a public health law framework. Nutrients 12, 1278. 32365952 10.3390/nu12051278 PMC7281994

[41] Mackay S , Sing F , Gerritsen S et al. (2020) Benchmarking Food Environments 2020: Progress by the New Zealand Government on Implementing Recommended Food Environment Policies & Priority Recommendations. Auckland: The University of Auckland.

[42] Backholer K & Sing F (2020) Controls on the Marketing of Food and Non-Alcoholic Beverages to Children in Thailand: Legislative Options and Regulatory Design. Bangkok: UNICEF Thailand.

[43] United Nations General Assembly (1989) Convention on the Rights of the Child. United Nations. https://www.unicef.org/sites/default/files/2019-04/UN-Convention-Rights-Child-text.pdf (accessed July 2019).

[44] World Health Organization (2018) Monitoring and Restricting Digital Marketing of Unhealthy Products to Children and Adolescents. Moscow: World Health Organization.

[45] Tatlow-Golden M , Jewell J , Zhiteneva O et al. (2020) Rising to the challenge: introducing protocols to monitor food marketing to children from the World Health Organization Regional Office for Europe. Obes Rev 22, e13212. 10.1111/obr.13212 34184400

[46] Truebridge N (2020) Health Officials want Beefed up Advertising Rules Amid Grim South Auckland Child Obesity Outlook. RNZ. https://www.rnz.co.nz/news/national/407574/health-officials-want-beefed-up-advertising-rules-amid-grim-south-auckland-childhood-obesity-outlook (accessed January 2020).

[47] Colmar B (2020) Children’s Media Use Survey 2020. https://www.nzonair.govt.nz/research/childrens-media-use-survey-2020/ (accessed January 2020).

[48] World Health Organization (2016) Tackling Food Marketing to Children in a Digital World: Trans-Disciplinary Perspectives. Copenhagen: WHO Regional Office for Europe.

[49] Kidd B , Mackay S , Swinburn B et al. (2021) AdHealth: a feasibility study to measure digital food marketing to adolescents through Facebook. Public Health Nutr 24, 215–222. 32878674 10.1017/S1368980020001561 PMC10195416

[50] Stewart T , Duncan S , Walker C et al. (2019) Effects of Screen Time on Preschool Health and Development. Wellington: Ministry of Social Development.

